# Dirt or type? Clarifying fungal nomenclature in the DNA age

**DOI:** 10.3897/imafungus.17.190622

**Published:** 2026-07-17

**Authors:** Vasco Fachada, Irina S. Druzhinina

**Affiliations:** 1 Royal Botanic Gardens, Kew, Kew Green, Richmond, Surrey, UK Royal Botanic Gardens, Kew Richmond United Kingdom

**Keywords:** Admixtures and gatherings, fungal dark taxa, ICN, Index Fungorum, MycoBank, specimenhood, taxonomy, typification

## Abstract

The rapid expansion of high-throughput sequencing and environmental DNA (eDNA) studies has led to the introduction of dozens of fungal names based exclusively on molecular data derived from environmental substrates such as soil, dung, or water. In several recent cases, authors and nomenclatural repositories have divergently treated such names as valid or invalid on the grounds that the designated types may or may not constitute specimens under the current wording of the International Code of Nomenclature for algae, fungi, and plants (ICN; Madrid Code). Here, we examine the nomenclatural basis of these treatments by analysing the relevant ICN provisions on specimens, gatherings, admixtures, and typification. We show that while a conservative curatorial approach is defensible under current practice and aligned with the ICN’s objective of nomenclatural stability, the Code does not explicitly address whether preserved environmental samples can serve as types when a taxon is evidenced solely by a molecular signal. We therefore argue that explicit clarification—through formal proposals to amend Chapter F and/or relevant general ICN provisions—is now urgently required to ensure consistent, transparent, and future-ready nomenclatural practice in the face of accelerating detection-driven discovery.

Graphical Abstract

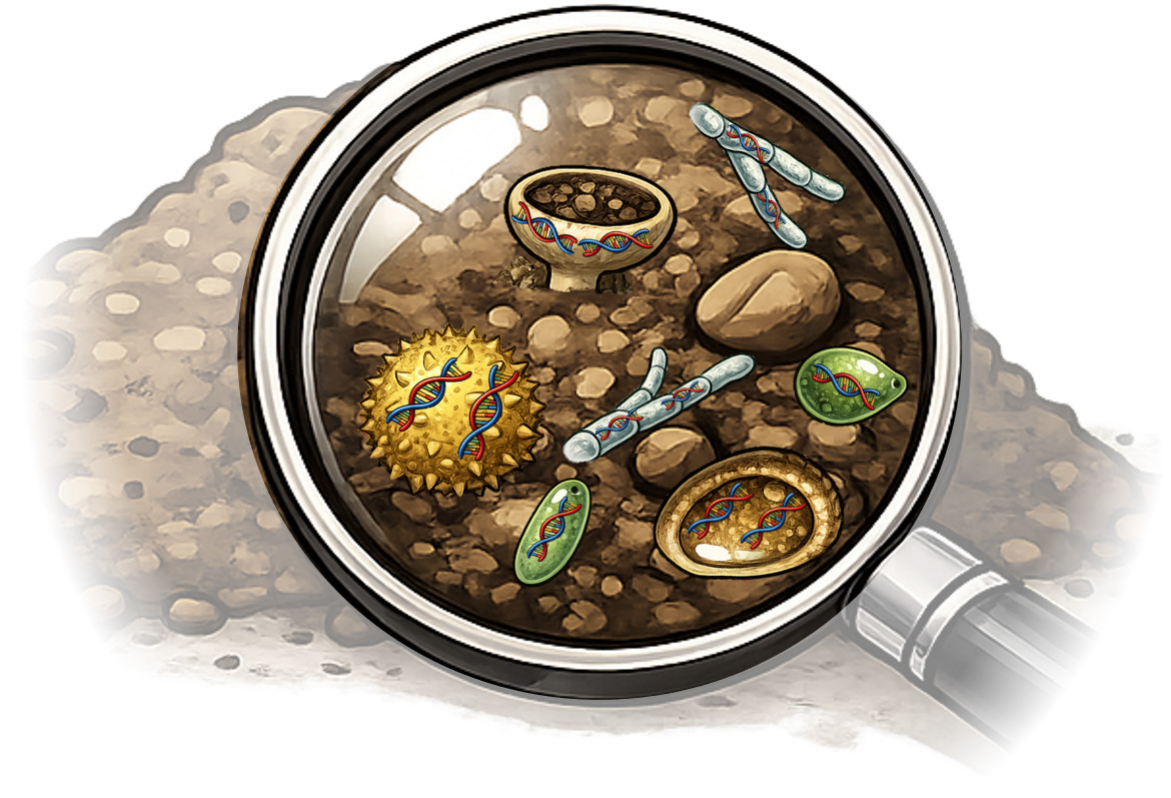

## Introduction

Advances in molecular biology have profoundly transformed fungal systematics, revealing extensive cryptic diversity and uncovering lineages that remain unknown from morphology or culture, herein called fungal dark taxa or FDT (Grossart et al. 2016; Selbmann et al. 2020; Lücking et al. 2021; Zhang et al. 2025). Environmental sequencing, in particular, has generated large numbers of phylogenetically distinct molecular operational taxonomic units that may represent previously undescribed taxa (Schadt et al. 2003; Hibbett and Taylor 2013; Tedersoo et al. 2014). This development has raised fundamental nomenclatural questions, especially concerning the nature of types and specimens when novel taxa are detected in complex environmental substrates (Ryberg and Nilsson 2018; Nilsson et al. 2023; Zhou 2024). Under the International Code of Nomenclature for algae, fungi, and plants (ICN, the Code), the application of names at the rank of genus and below is anchored to a nomenclatural type, which is ordinarily a preserved physical specimen representing a single taxon, designated at the time of valid publication.

The status of DNA sequence data in fungal nomenclature has been the subject of sustained scholarly debate, particularly in relation to typification, interpretation of specimenhood, and the long-term stability of names (Lehtonen and Thiele 2023; Thiele et al. 2023; Turland 2025). A series of coordinated contributions have examined both the opportunities and risks associated with sequence-based, voucherless taxonomy, highlighting concerns over reproducibility, traceability, and nomenclatural stability, while also acknowledging the transformative potential of high-throughput sequencing for documenting fungal diversity (Lücking and Hawksworth 2018; Lücking et al. 2018; Zamora et al. 2018). Broader syntheses have further emphasized the tension between eDNA-based diversity and the requirements of traditional nomenclatural frameworks, underscoring the need for coherent, community-driven standards for integrating molecular data into fungal taxonomy (Wu et al. 2019; Lücking et al. 2020, 2021).

The latest episode intensifying this debate began with the attempted introduction of 30 species of FDTs, representing previously unnamed higher-level lineages detected in soil, water, sediment, and dung, together with more than 160 names based on eDNA sequences (Tedersoo et al. 2025), which were subsequently treated as invalid in their records by nomenclatural repositories, notably Index Fungorum (IF), MycoBank (MB), and Fungal Names (FN) (as of 30 January 2026). In these cases, the central issue is whether an environmental sample, such as soil or dung, can constitute or contain a specimen under the ICN, and therefore whether the requirement for type indication has been met. These actions have prompted renewed debate among fungal taxonomists and nomenclatural specialists, reflecting divergent interpretations of the Code, rather than disagreement over its objectives.

The aim of the present forum paper is not to argue whether eDNA-based taxa should or should not be accepted under the ICN in the future. Instead, we examine a narrower but crucial question: whether the current wording of the Code unambiguously mandates the invalidation of names typified by environmental samples, or whether more than one interpretation remains compatible with the existing rules. By analysing the relevant articles and the reasoning reflected in recent repository treatments of such names, we identify points at which the Code, current practice, and community expectations do not yet align fully. We therefore argue that formal clarification is required, not only to resolve individual disputed cases, but to ensure that fungal nomenclature remains governable, consistent, and transparent as detection-driven discovery continues to accelerate.

## Ropeless anchors, drifting boats

The main purpose of a type is to anchor a species concept to a name. Without a type, any name will ultimately drift away from the original concept, aimlessly and perpetually, rendering the name of little practical value to the scientific community. Consequently, the formal designation and curation of such anchors are of the utmost importance for taxonomists and therefore required by the Code (Turland et al. 2025: Art. 7.1 and 7.2).

Nonetheless, the purpose of a type should arguably extend beyond its mere legal need to exist, particularly in the context of modern taxonomy (Durkin et al. 2020; Aime et al. 2021; Yurkov et al. 2024). It should be as information-dense as possible, and importantly, this information should be continuously accessible. These conditions are not explicitly considered by the Code at the moment of typification, and their absence can result in the same drifting of names as if no typification occurred in the first place. Beyond whether DNA itself should or could already be considered a type, it is crucial to acknowledge the upstream purposefulness of a type.

This problem is well illustrated by yeasts and other fungi commonly studied in culture, where revision, authentication, physiological comparison, and modern molecular reassessment often depend on access to viable ex-type or reference cultures, even though such cultures may not themselves have formal nomenclatural status under the current Code (Yurkov et al. 2024).

While DNA typification could potentially provide infinitely lasting ropes connecting anchors to names, it is also fair to fear it may, without careful regulation, generate rather thin connecting strings between the same anchors and names, literally as thin as DNA strands themselves (Lehtonen and Thiele 2023; Thiele et al. 2023). In any case, a type should ideally facilitate the revisiting, authentication, comparison, and expansion of a species concept.

## Probing the Code: stress-testing the rules

Several recent and historical cases illustrate how the ICN has been tested at the boundary between emerging discovery practices and established nomenclatural concepts. Although these cases may be perceived as attempts to circumvent the Code, they can also be understood as practical stress-tests that expose where broadly framed, method-neutral provisions encounter biological and technological scenarios not explicitly anticipated when the relevant articles were drafted.

An instructive historical example is *Lawreymyces palicei* Lücking & B. Moncada (*Corticiales*, *Basidiomycota*), where the authors proposed that a representation of a DNA sequence could satisfy the illustration route available under the Code at that time (McNeill et al. 2012; Lücking and Moncada 2017). Subsequent editions of the ICN, however, explicitly deemed those particular representations of DNA sequences as “not depicting features of the organisms”, and therefore, not illustrations under Art. 6.1, thereby closing this route for future names (Turland et al. 2018, 2025). Nevertheless, the distinct theoretical question was left open: how realistic a DNA depiction must be to qualify as a “feature of an organism”, and therefore as an illustration under the Code?

More recently, Tedersoo and colleagues attempted to formally describe multiple FDTs based on environmental samples, proceeding to voucher and designate holotypes from these collected materials (Tedersoo et al. 2025). This approach represents a deliberate effort to operate within existing ICN requirements, including the requirement for physical type material, while making explicit the practical challenges posed by taxa that are detectable only through molecular evidence. Comparable circumstances are not unprecedented: *Piromyces cryptodigmaticus* (*Neocallimastigales*, *Neocallimastigomycota*) was described under similar conditions, with typification anchored to preserved environmental material (cow manure) from which diagnostic DNA sequences were derived (Kirk and Griffith 2021). These cases illustrate that the underlying difficulty is not the absence of a type indication, but the interpretation of whether the designated material can satisfy the definition of a specimen under Art. 8.

In the latter examples (Kirk and Griffith 2021; Tedersoo et al. 2025), DNA sequences served as the basis for diagnosis and delimitation, while the cited type material was a physical environmental sample. The ICN has not explicitly pronounced on whether preserved environmental samples that yield only molecular evidence can constitute specimens for typification (Turland et al. 2025), and the conformity of such cases therefore remains open to interpretation. This openness reflects the fact that the relevant provisions were drafted before the present issue arose, namely the attempted description of fungi from environmental samples in which the taxon is evidenced only by molecular data.

Viewed in this light, recent exercises in naming fungi from environmental samples should be seen as exposing an ontological tension inherent to modern taxonomy: the increasing need to name and communicate about taxa that are biologically real but difficult or impossible to characterise using traditional morphological or cultural criteria (Franz 2011). The relevant question is therefore not whether such work “challenges” the Code, but whether the current wording of the Code already provides sufficient clarity to accommodate these cases, or whether explicit guidance is now required.

This distinction is critical. Stress-testing the Code through real-world applications is a legitimate and historically productive mechanism by which ambiguities are revealed and, where necessary, resolved through formal governance processes. Whether names such as *Piromyces cryptodigmaticus*^1^ or *Curlevskia holarctica*^2^ (*Curlevskiales*, *Curlevskiomycota*) are ultimately deemed valid under the ICN is less important, at this stage, than recognising that their treatment depends on interpretation rather than on unequivocal rules. How such interpretations may be stabilised or revised through ICN governance is addressed in the following sections.

## Bedrock of typification: most relevant provisions of the ICN

### The ontology of a specimen

**Figure 1.**
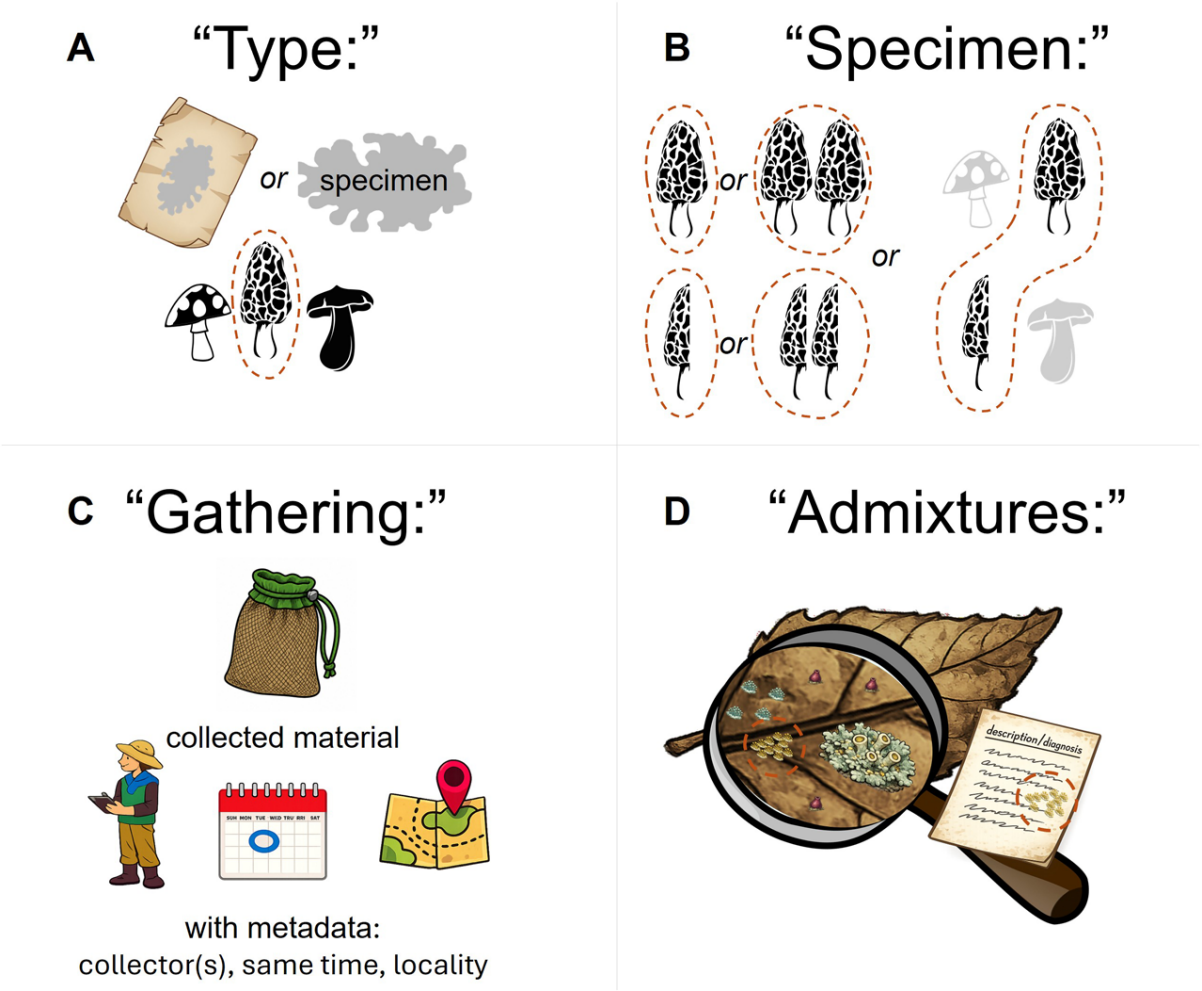
Schematic interpretation of ICN provisions relevant to typification and admixtures. **A**. A nomenclatural type is a specimen or an illustration. **B**. For the purposes of typification, a specimen is a gathering, or part of a gathering, of a single species or infraspecific taxon, disregarding admixtures (Art. 8.2). **C**. A gathering is collected material obtained by the same collector(s), at the same time, from a single locality, and presumed to be of a single taxon (Art. 8.2). **D**. If material associated with a type is found to contain parts belonging to more than one taxon, the name remains attached to the part that corresponds most nearly with the original description or diagnosis (Art. 9.14).

Article 8.2 of the ICN defines a specimen, for the purpose of typification, as “a gathering, or part of a gathering, of a single species or infraspecific taxon, disregarding admixtures (see Art. 9.14)” (Fig. 1). A specimen may consist of a single part, multiple parts, or the whole of one or more individual organisms, and is usually preserved on a single herbarium sheet or in an equivalent preparation such as a box, packet, jar, or microscope slide.

The Article also clarifies the use of the term ‘gathering’, specifying that it refers to material collected by the same collector(s), at one given time and from one locality, and presumed to be of a single taxon (Fig. 1). Importantly, this presumption is not a guarantee that the gathering truly contains only one taxon, as the ICN, under Art. 9.14, explicitly recognises that admixtures may later be discovered, particularly in the context of typification. Furthermore, under Art. 8.2, a type need not consist of the entire gathering, as a specimen may correspond to only part of a gathering (Fig. 1).

Two aspects of this framework are particularly relevant. First, the ICN explicitly accommodates the presence of admixtures, provided that the specimen can still be treated as representing a single taxon for typification purposes. Second, the ICN does not specify the nature of the admixtures or substrate on which, or within which, the organism occurs. There is no explicit distinction between plant material, animal substrates, soil, dung, or other matrices, provided that the requirements of specimenhood are met under Art. 8.2 (Turland et al. 2025).

### Admixture and identity

Article 9.14 addresses situations in which material associated with a type contains elements belonging to more than one taxon (Fig. 1). In such cases, the name is to remain attached to “the part (specimen as defined in Art. 8.2) that corresponds most nearly with the original description or diagnosis.” This provision establishes a rule of selective name attachment rather than automatic invalidation, thereby supporting nomenclatural continuity when type material is later found to be mixed.

In practice, Art. 9.14 has long been applied to a wide range of mixed or host-associated collections, including host–parasite systems, lichenized and lichenicolous fungi, endophytes, and microfungi occurring on or within heterogeneous substrates (Hawksworth 2003; Gómez-Zapata et al. 2024; Turland et al. 2025). Such situations are routine in mycological practice and reflect the biological reality that many fungi are encountered as components of complex assemblages rather than as isolated, discrete entities. Lichenized fungi are instructive in this respect. For this case, biological association or admixture is not itself the nomenclatural problem, provided that the name-bearing fungal component can be related to observable thalli, reproductive, anatomical, or otherwise directly visible features. The difficulty becomes sharper when an additional sequence-detected fungus is inferred from the same material but cannot be optically localised as a corresponding organismal structure, as illustrated by the *Lawreymyces* case (Lücking and Moncada 2017).

### Fixing the name

Article 40.1 states that publication of the name of a new taxon at the rank of genus or below is valid only when the type of the name is indicated. The Article itself does not define what constitutes an acceptable specimen; rather, it presupposes that the indicated type conforms to the definition of a specimen as set out in Art. 8. In this sense, Art. 40.1 establishes a necessary procedural requirement for valid publication, while the substantive criteria for specimenhood are governed elsewhere in the Code.

Failure to indicate a type results in invalid publication of the name, irrespective of the quality, quantity, or sophistication of the descriptive or diagnostic information provided (Turland et al. 2025).

Accordingly, divergent readings over the validity of names based on environmental samples do not arise from a failure to indicate a type per se, but from differing interpretations of whether the designated material satisfies the definition of a specimen under Art. 8.2.

## From sample to status: recent nomenclatural decisions

### Repository treatments and their nomenclatural basis

**Figure 2.**
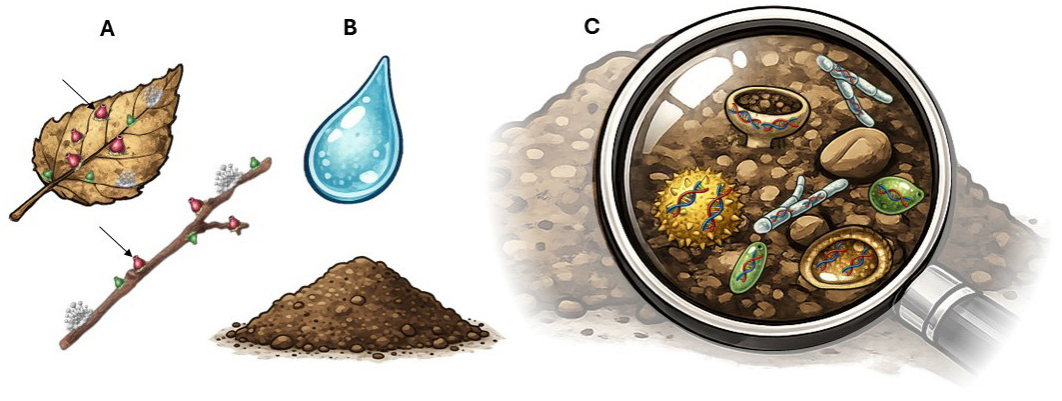
Mixed substrates and localisation of the putative name-bearing element. **A**. In conventional microfungal typification on substrates such as leaves or twigs, several organisms may be present, but the focal fungus can usually be visualised, localised, and related directly to the description or diagnosis. Arrows indicate examples of localised putative name-bearing elements. **B**. Environmental substrates such as water or soil may contain biological material that is less readily localised as a discrete organismal structure. **C**. Soil substrate containing multiple biological elements, including spores, hyphae, sporophores, and recoverable DNA. The figure illustrates the practical distinction between easily optically localisable fungal evidence and hardly or non-optically localisable fungal evidence in mixed substrates.

In the cases discussed above (Tedersoo et al. 2025), where some recognized repositories have treated names based on environmental samples as invalidly published, the underlying reasoning has generally been that samples such as soil, dung, or similar substrates cannot readily be treated as specimens representing a single taxon within the meaning of Art. 8.2^3^. This position, more than denying that such samples constitute gatherings in a procedural sense, emphasises the practical difficulty—or impossibility—of identifying or delimiting a specific physical part of the sample that can function as the name-bearing element, particularly when no morphological structures, cultures, or microscopic preparations are provided and the diagnosis is exclusively molecular (Fig. 2).

From this perspective, Art. 9.14 is considered inapplicable, because no identifiable “part” of the type material can be unequivocally linked to the original description or diagnosis. Environmental samples are therefore treated as distinct from more traditional substrates, such as leaves or twigs bearing visible fungal structures, in which a name-bearing element can be conceptually and optically localised despite the presence of admixtures (Fig. 2).

Interestingly, at the time this work was being prepared, all recognized repositories treated *C. holarctica* and the other species described by Tedersoo et al. (2025) as invalidly published under Art. 8.2, whereas only MB treated *P. cryptodigmaticus* (Kirk and Griffith 2021) as invalidly published. The latter assessment differed from the treatment adopted by IF and FN, despite an ongoing scrutiny concerning the adequacy of the description or diagnosis of *P. cryptodigmaticus* (Tripp and Lendemer 2012). The divergent repository treatments illustrate that the application of the Code in complex cases is not always consistent, even among recognized nomenclatural authorities. Mechanisms exist within the ICN both for resolving questions concerning particular names and for amending provisions whose general application remains unclear (Box 1). The contrasting treatment of these cases underscores that assessments of valid publication may depend not only on the material designated as type, but also on how Arts. 8 and 9 are interpreted in relation to biologically complex material.

**Box 1.** Governance and routes for advice or amendment in fungal nomenclature.

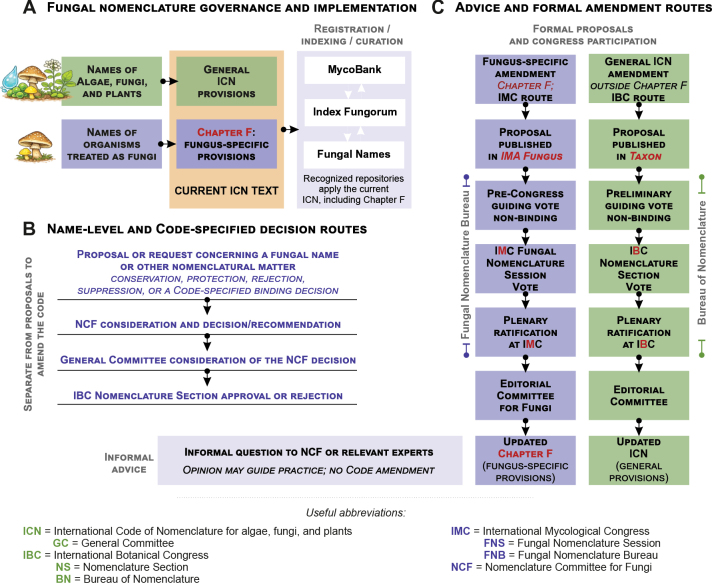
**A**. Governance and implementation layers relevant to fungal nomenclature under the International Code of Nomenclature for algae, fungi, and plants (ICN). Names of organisms treated as fungi are governed by (i) the general provisions of the ICN together with (ii) the fungus-specific provisions in Chapter F. Thus, fungal names are subject both to the general ICN governance framework associated with the International Botanical Congress (IBC), its Nomenclature Section, and the Editorial Committee, and to the fungus-specific governance framework associated with the International Mycological Congress (IMC), its Fungal Nomenclature Session, and the Editorial Committee for Fungi. Successive editions of the ICN are conventionally referred to by the locality of the IBC at which their amendments were adopted; for example, the Shenzhen Code was the edition based on decisions of the 2017 IBC in Shenzhen (Turland et al. 2018), whereas the Madrid Code incorporates amendments adopted at the 2024 IBC in Madrid (Turland et al. 2025). Chapter F may also be cited by the locality of the IMC at which its changes were adopted when it is published separately, as in the San Juan Chapter F (May et al. 2019). When the timing of an IMC and IBC permits, Chapter F revisions may instead be incorporated into the next full Code. In the 2024 cycle, the Chapter F changes adopted at IMC12 in Maastricht were incorporated into the Madrid Code rather than published as a separate edition of Chapter F (May et al. 2024; May and Bensch 2026).
Registration of fungal nomenclatural novelties through recognized repositories was introduced in the Melbourne Code, effective from 1 January 2013, and is now treated in Chapter F as Art. F.5 (McNeill et al. 2012; May 2020; Turland et al. 2025). Under Art. F.5.3, the Nomenclature Committee for Fungi (NCF) has the power to appoint one or more localized or decentralized, open and accessible electronic repositories to accession the required information and issue identifiers, to cancel such appointments, and to set aside the registration requirements if the repository mechanism fails, with such decisions subject to ratification by a subsequent IMC (Turland et al. 2025). The currently recognized repositories are Fungal Names (https://nmdc.cn/fungalnames/; China), Index Fungorum (http://www.indexfungorum.org/; United Kingdom), and MycoBank (https://www.mycobank.org/; Netherlands); their appointment by the NCF was ratified by the 10^th^ International Mycological Congress (Art. F.5, Ex. 1; Turland et al. 2025). These repositories operate at the implementation level: they accession the required information, issue identifiers for fungal nomenclatural novelties and for type designations covered by Art. F.5.4, apply the current ICN, including Chapter F, in registration, indexing, and curation workflows, and may coordinate and synchronize records within this framework.
**B**. Name-level and Code-specified decision routes. Name conservation, protection, rejection, suppression, and Code-specified binding-decision procedures follow the General Committee and relevant specialist-committee routes and are distinct from proposals to amend the wording of either the general ICN provisions or Chapter F (May and Bensch 2024). These procedures can resolve particular nomenclatural cases or Code-specified questions, but they do not provide a general route for amending the Code or for issuing a broad binding interpretation on whether environmental samples constitute Code-compliant type material.
**C**. Routes for advice or amendment when uncertainty relevant to fungal nomenclature arises. Informal questions may be addressed to the Nomenclature Committee for Fungi (NCF) or other relevant experts; such opinions may guide practice, but they do not amend the Code and should be distinguished from Code-specified binding-decision routes (May 2020; May and Bensch 2024). Formal amendment of general ICN provisions follows the relevant call-for-proposals cycle and requires publication of a proposal in Taxon (Wiley), synopsis and commentary, a preliminary Guiding Vote among eligible voters, consideration by the IBC Nomenclature Section, ratification by the IBC plenary session, and incorporation by the Editorial Committee (Turland and Wiersema 2024; Turland et al. 2024a, 2024b, 2026). Formal amendment of fungus-specific provisions follows the relevant call-for-proposals cycle and requires publication of a proposal in IMA Fungus (Pensoft Publishers), synopsis and commentary, a non-binding pre-Congress Guiding Vote among eligible voters, consideration by the IMC Fungal Nomenclature Session, ratification by the IMC plenary session, and incorporation into Chapter F by the Editorial Committee for Fungi, with consultation with the Editorial Committee as required (May and Miller 2018; May 2020; May and Bensch 2024; May et al. 2024). Outcomes of the IBC Nomenclature Section and IMC Fungal Nomenclature Session are reported after the relevant congresses; accepted amendments are then incorporated into the next edition of the ICN or, for fungus-specific changes, into Chapter F, either as a separate Chapter F edition or as part of the next full Code when timing permits (May et al. 2024; Turland et al. 2024b).
The Fungal Nomenclature Bureau administers the Fungal Nomenclature Session and pre-Congress Guiding Vote, while the NCF provides expert opinions on proposals. IMA Fungus is the journal of the International Mycological Association (IMA), under whose auspices International Mycological Congresses are held, and is the publication venue for formal proposals to amend Chapter F, synopses of such proposals, and related fungal nomenclatural communications. For convenience, relevant abbreviations are also given below the figure.

### Alternative interpretations

Admittedly, one could argue that this reasoning may go beyond what is articulated in the Code. Specifically, it may be stated that Art. 8.2 allows admixtures and does not explicitly require the taxon to be morphologically visible or palpably separable at the time of typification. From this viewpoint, the decisive factor is whether the organism was physically present in the designated type material, rather than whether it can be visually localised or delimited within that material at the time of typification (Fig. 2).

Under this interpretation, recovering a DNA sequence from an environmental sample can be taken as evidence that the organism occurred in, and was represented within, that sample, and thus that the sample could be interpreted as containing material referable to the specimen (Fig. 2). The Code does not explicitly state that the relevant material must be recognisable by morphological means, nor that recognition must occur without recourse to analytical techniques. In the same way one may need an optical microscope to detect a spore belonging to a given taxon, one may also need molecular methods to detect taxon-attributable DNA fragments in preserved material. Although *Lawreymyces*-like DNA representations may not be currently permitted as illustration types, it could be argued that recoverable taxon-attributable DNA within the preserved material may be considered a name-bearing component under Articles 8.2 and 9.14 (Fig. 2). This interpretation is possible and could be considered literal, as nothing in the Code mandates on the scale between gatherings and parts of gatherings (Turland et al. 2025).

### Interpretation versus rule: the Code’s ambiguity on specimenhood

Taxonomically agnostic, the Code’s primary concern is the application, stability, and communicability of names, rather than the regulation of methods of taxon discovery. Taxonomy, by contrast, is not governed through an equivalent legislative framework, but through evolving evidentiary standards, peer review, specialist expertise, and community practice. Nonetheless, typification sits at the interface between nomenclature and taxonomy, which is why the present friction arises (Thomson et al. 2018; Lehtonen and Thiele 2023; Turland et al. 2025: Art. 7.1 and 7.2).

The contrasting positions outlined above illustrate that the ICN does not provide an explicit answer to the question of whether environmental samples can serve as material eligible for typification. Terms such as “specimen” and “gathering” are defined only in a broad sense, leaving room for interpretation when applied to modern molecular practices and biological realities that were not envisaged when the relevant articles were drafted.

Importantly, the ICN does not state that a specimen must include visible structures, a culture, or a physically isolatable organism. Nor does it state that mixed or environmental substrates are excluded. At the same time, the ICN does not explicitly endorse the use of environmental samples as type-bearing material when no physical manifestation of the organism can be demonstrated beyond a DNA sequence. As a result, decisions to accept or reject such names necessarily involve interpretative judgement in applying Art. 8.2 to cases not explicitly anticipated by the wording of the Code. How such interpretative judgments can be formalised, stabilised, or revised within the ICN governance framework is outlined in Box 1.

In this context, the recent treatment of certain names as invalid in recognized repositories should be understood as reflecting a conservative interpretation of the Code in circumstances where the rule is not explicitly articulated. An environmental sample such as soil, water, sediment, or dung may satisfy some procedural elements associated with a gathering, such as collection by a collector at a defined time and place. Whether it can also satisfy the Art. 8.2 requirement that a gathering be presumed to be or contain a specimen of a single taxon for typification remains a distinct interpretative question under the current wording of the Code. While repository caution is understandable, especially given the potential proliferation of poorly anchored names, divergent curatorial assessments may create inconsistency when different actors apply different readings of the same Articles. Where the basis for repository status assessments is not sufficiently explicit, or where disputed assessments are difficult to revisit, user confidence in the registration and curation system may be weakened.

The breadth of these definitions is not a defect in itself: it reflects the Code’s role as a stability framework intended to accommodate diverse biological realities and evolving methods.

## Ripples in the system: implications for nomenclatural practice

The current situation places nomenclatural repositories in a difficult position. On the one hand, they are expected to apply the Code consistently and to safeguard nomenclatural stability. On the other hand, they must confront novel cases that fall outside the scenarios explicitly addressed by the Code. In the absence of clear guidance, status assessments may be criticised either for being too permissive or too restrictive.

A cautious approach, such as refraining from accepting environmental samples as type-bearing material, may be defensible as an interim measure. However, it should be recognised as an interpretative position adopted under conditions of uncertainty, rather than as a direct and unavoidable consequence of ICN wording. Explicitly acknowledging this distinction would enhance transparency and foster constructive discussion within the community. Occasional disagreement among repositories should therefore not necessarily be viewed negatively: it may help identify underspecified areas of the Code and stimulate formal pathways toward consensus-building. At the same time, because repository status assessments are highly influential in practice, sustainable progress would benefit from transparent curatorial criteria, efficient mechanisms for revisiting disputed assessments, and broader engagement with taxonomic expertise. Complementary resources, including sequence-based infrastructures such as UNITE (Abarenkov et al. 2024) and global metabarcoding occurrence resources such as GlobalFungi (Větrovský et al. 2020), may also play an important role in documenting and communicating molecular diversity. However, they do not issue nomenclatural identifiers and therefore do not perform the function of recognized repositories under the Code.

For the time being, different groups of mycologists working with FDT may be unsure how to proceed with their research, whether they should produce, hold, discard or publish eDNA data for taxonomic purposes. Concerningly, in an era of accelerating species detection, such uncertainty does not remain local or temporary, but scales rapidly across datasets, repositories, and research programmes.

Time will tell whether the approach adopted by Tedersoo et al. (2025), based on labelled environmental samples designated as types—an approach intended to provide a physical voucher for otherwise voucherless molecular taxa—will be accepted or regarded as another attempt to circumvent the Code, as in *Lawreymyces palicei* (Lücking and Moncada 2017). Regardless of one’s position, these cases should be recognised as good-faith attempts to operate within existing rules while making a practical problem visible to the community.

## Holding the type and holding to taxonomic tradition

It seems we may be traversing a general transformation, both in taxonomy and in the taxonomist and their skillset, as the traditional anatomy-focused taxonomist dwindles (Durkin et al. 2020; Lofgren and Stajich 2021; Zhou 2024; Simões et al. 2025) and gives way to, or is complemented by, the modern polyphasic and molecular taxonomist (Gannibal 2022; Kim 2024; Bernard 2025; Li et al. 2025).

Although molecular fungal taxonomy has existed for several decades, and mycologists have proposed ways to formally describe voucherless FDT (Hibbett et al. 2011; Ryberg and Nilsson 2018; Lücking et al. 2021; Thiele et al. 2023; Zhou 2024), the taxonomic community has resisted DNA-based typification of FDT (Lücking et al. 2021; Lehtonen and Thiele 2023; Zhou 2024).

A prevailing view in the community seems to be the unwritten expectation for the fungal specimen itself to be not only physical, but also palpably recognisable in some form. Conceptually, it can be argued that DNA is not less physical than a spore or a hypha, both are material and observable with distinct methods, the difference lying not in materiality, but in optical describability. Thus, the willingness of taxonomists and nomenclaturists to accept a leaf densely populated with mingling taxa as suitable for typification, but not an equally populated jar of soil, may reflect tradition more than the actual wording of the Code (Fig. 2).

Such reluctance may reflect the central role that morpho-anatomical expertise has historically played in anchoring fungal names to tangible biological context. The present debate therefore mirrors a broader structural transition in systematics, as detection increasingly precedes full characterisation, raising legitimate questions about the depth of contextualisation required at the moment of typification and the role of modern taxonomists.

**Figure 3.**
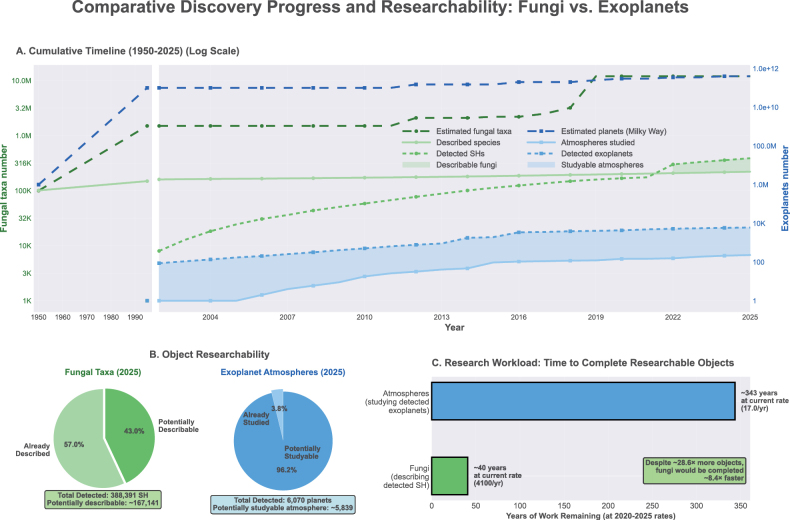
Comparative discovery, detection, and researchability trajectories of fungi and exoplanets. **A**. Cumulative timelines illustrating estimated total objects, detected objects, and in-depth studied objects for fungi (left, green axis) and exoplanets (right, blue axis). Curves are plotted on logarithmic scales with axis labels reflecting raw values. For fungi (green), lines represent estimated global fungal diversity (long dashed line), detected species hypotheses (SHs; operationally defined at a 1% threshold) from sequence data (short dashed line), and formally described taxa (solid line). For exoplanets (blue), lines represent estimated planetary abundance in the Milky Way (long dashed line), detected exoplanets (short dashed line), and planets with atmospheres studied in detail (solid line). Shaded areas indicate the gap between detection and in-depth study, here referred to as researchability (i.e. potentially describable or studyable objects). **B**. Object researchability in 2025, contrasting the proportion of already described or studied objects with those detected but not yet investigated in depth, for fungal taxa and exoplanet atmospheres, respectively. **C**. Estimated research workload based on 2020–2025 discovery and study rates, showing the projected time required to complete research on currently researchable objects. Despite the much larger absolute number of potentially researchable exoplanets, newly detected fungal taxa are substantially more accessible to in-depth study and therefore likely to be formally described at a markedly faster rate. Modelled based on data from (Gardes and Bruns 1993; Cassan et al. 2012; Robert et al. 2013; Hawksworth and Lücking 2017; Wu et al. 2019; Hyde et al. 2020; NASA Exoplanet Science Institute 2020; Kirk and Griffith 2021; Nilsson et al. 2023; Abarenkov et al. 2024, 2025; Christiansen et al. 2025; NASA 2026; Exoplanet Team 2026).

Unsettlingly, this is already the reality in astronomy, where exoplanetologists routinely assign systematic, permanent identifiers to newly detected planets and stars, only later addressing their physical characterisation and interpretation. Crucially, however, the comparison is asymmetric: for each predicted fungal species on Earth, there are tens of thousands of predicted planets in the Milky Way alone, and the technological barrier to studying fungal taxa is far lower than that for exoplanets (Hawksworth and Lücking 2017; Boettner et al. 2024; Bernard 2025). Consequently, not only is the absolute number of detected and described fungal taxa much higher, but their researchability is also substantially greater (Fig. 3), although any projected future rates remain hypothetical and will depend on taxonomic capacity, automation, and the standards adopted for DNA-only taxa. The acceleration of fungal discovery also makes use of automated identifiers, through UNITE’s species hypothesis (SH). Unlike in astronomy, these are configurable and shifting concepts, often including several taxa (Abarenkov et al. 2024). Put together, this underscores the continuing and highly relevant role of the modern fungal taxonomist in defining, contextualising, and understanding taxa, regardless of the accelerating pace of name introduction.

## Clearing the mud: towards a more explicit concept of specimenhood

Building on the ambiguities outlined above, clarification cannot remain purely interpretative. Beyond refining existing readings of Arts. 8 and 9, several procedural avenues may be considered. Formal proposals could explicitly address the status of environmental samples and DNA-derived evidence, beginning with a precise definition of what constitutes an “environmental sample” in nomenclatural terms. At present, the Code does not distinguish between substrates such as leaves, wood, dung, soil, or water; yet much of the disagreement arises from implicit assumptions about their equivalence or non-equivalence. A definition grounded in collection procedure and material composition would enhance consistency and reduce interpretative drift.

Alternatively, a clearly delimited interim category for sequence-based fungal designations could be explored, pending broader consensus and formal Code action (see Box 1 for amendment routes). A comparable mechanism has already been proposed through the postnominal *nom. seq*. (*nomen sequentium*), intended for sequence-based designations where no specimen or illustration is available to serve as a Code-compliant nomenclatural type and to indicate that such designations are not validly published under the current ICN (Lücking et al. 2018; May and Hawksworth 2024). Such a mechanism would allow the community to record, discuss, and evaluate DNA-based taxonomic entities in a transparent and controlled manner, without granting them immediate equivalence to Code-compliant names or destabilising the traditional specimen-based framework. The most recent Chapter F proposal to introduce this mechanism was considered at IMC12 but was not accepted (May et al. 2024). The procedures and timetable for proposals to amend Chapter F at IMC13 have now been published, with proposals due by 31 December 2026 (May and Bensch 2026), providing a current formal route for reconsidering such mechanisms.

If, conversely, DNA-based typifications were to be incorporated within the existing structure of the Code, safeguards would be required to preserve descriptive depth and avoid erosion of integrative taxonomy (Thines et al. 2018; Zamora et al. 2018). One possibility would be to restrict DNA-based typification to cases in which preservation of a traditional physical specimen, or isolation and maintenance of a pure culture, is technically unfeasible, and to require explicit justification that conventional typification could not reasonably be achieved (May and Hawksworth 2024). Such restrictions would not privilege morphology over nucleotides or genes, but would aim to ensure that DNA-based naming remains a last resort rather than a default strategy.

Behind the present debate about environmental samples or DNA typification, at its core lies the fundamental issue: the operational meaning of “specimen” under the ICN. Regardless of whether the community ultimately accepts or rejects DNA-based typification, clarification is required on several points. Below we list critical nodes of ambiguity that should be addressed by the community when drafting Code-changing proposals:

1. Whether a specimen must be detectable only at the time of typification. Or rather, can or must it be detectable at any later time.
2. Whether detectability must involve direct morphological observation, including light or electron microscopy, or whether molecular and other analytical methods can also be sufficient or even mandatory. Intermediate solutions like the already proposed fluorescent *in situ* hybridization (FISH) should be addressed as well (Jones et al. 2011; Lücking and Hawksworth 2018).
3. How to realistically referee points a) and b).
4. Whether the type-bearing gathering must be physically integral and undivided like a leaf and a twig, or if it may be easily dispersed like soil, water and many leaves.
5. How distributed or non-visually localisable biological evidence should be treated under Arts. 8 and 9.

In this context, minor but precise adjustments to wording may already significantly enhance clarity. For example, Article 9.14 currently states: “When a type (herbarium sheet or equivalent preparation) contains parts belonging to more than one taxon…”. This phrasing conflates the actual type with its physical container, as by definition the type is a specimen belonging to only one taxon, not the curatorial material containing it. Reformulating this provision to clarify that the herbarium sheet or preparation may contain taxa in addition to the type-bearing specimen would more accurately reflect the logic of Art. 8.2 and reduce confusion in cases involving complex substrates.

Finally, the *Lawreymyces* case indicates that the status of illustrations may require further clarification where molecular features are invoked (Lücking and Moncada 2017). At present, the Code does not precisely define the informational threshold required for an illustration to constitute a “depiction of a species’ feature” under Art. 6.1, leaving room for divergent interpretations as imaging and molecular localisation technologies continue to evolve. One possible approach would be to explicitly define the minimum informational content required for an illustration in the context of molecular features, so that taxonomists are not left to infer adequacy on a case-by-case basis. Alternatively, the Code could restrict the use of illustrations as types to historical taxa while excluding their use for newly described taxa. Although such a restriction may appear censorious, it could prevent prolonged debates over progressively shifting criteria. This is particularly relevant given that the ability to depict DNA and its nucleotides at increasingly finer levels of detail is largely a retracting technological limitation rather than a stable conceptual boundary.

None of the measures above presuppose a particular outcome regarding the validity of DNA-based types. What they share is the potential to reduce uncertainty and help ensure that decisions by all stakeholders are seen as grounded in collectively agreed rules rather than individual interpretation.

## Conclusions for a molecular future

The ICN, in its current form, does not resolve whether environmental samples can serve as, or contain, specimens for the purpose of typification. Recent treatments of certain names as invalid in the recognized repositories, including IF, MB, and FN, apparently reflecting NCF-level consideration or advice, represent a conservative and defensible interpretation, but not one that is explicitly required by the Code. With environmental sequencing becoming increasingly prominent in fungal systematics, this ambiguity now requires attention. In view of the risk of disengagement, and even possible fragmentation of practice within parts of the community, formal clarification of the relevant articles is needed to ensure consistency, transparency, and confidence in fungal nomenclature (Box 1).

DNA is a physical molecular entity and an integral component of living organisms. When recoverable from preserved material, it therefore constitutes part of the physical evidence contained in that material. From an ontological perspective, molecular detectability and microscopic detectability represent different technological modalities of observing an equally physical reality (Fig. 2). Consequently, the distinction between DNA-based detection and morphological detection is methodological rather than material. The difficulty lies not in the physical nature of DNA, but in the absence of explicit provisions in the ICN for treating distributed molecular signals as constituting evidence of specimenhood, very much in the same way as optical signals are.

Nevertheless, the routine facilitation and broad adoption of DNA-based typification have the potential to diminish the quality of taxon descriptions and the depth of species concepts, a trend that may be reinforced by decreased numbers of mycologists trained in classical taxonomy. Given the realism of this pitfall, urgent but careful debate and action are in demand. What is at stake is not the status of soil or sequences, but the future coherence of biological naming in an era where organisms are increasingly detectable before they are understood, much like celestial objects (Fig. 3). In the DNA age, the practical question is no longer whether fungi occur in complex substrates, but whether nomenclatural governance is prepared to anchor names to evidence that may be molecular before it is morphological.

The question is therefore not whether fungal nomenclature will be confronted with accelerating, detection-led discovery, but whether its governance framework will be sufficiently prepared to absorb this acceleration without loss of coherence, transparency, and trust.

## Footnotes

see Piromyces cryptodigmaticus name Index Fungorum page https://www.indexfungorum.org/names/NamesRecord.asp?RecordID=551201.

see Curlevskia holarctica name Index Fungorum page https://www.indexfungorum.org/names/NamesRecord.asp?RecordID=859037.

see for example the Nomenclatural and Editorial comments and Typification details in Index Fungorum for Curlevskia holarctica Tedersoo, in Tedersoo, Hosseyni Moghadam, Panksep, Prins, Anslan, Mikryukov, Bahram, Abarenkov, Kõljalg, Esmaeilzadeh-Salestani, Pawłowska, Wurzbacher, Ding, Alkahtani & Nilsson, MycoKeys 124: 57 (2025) at https://www.indexfungorum.org/names/NamesRecord.asp?RecordID=859037: Nomenclatural comment: Nom. inval., Arts 8.2, 9.1 (Madrid). Editorial comment: Type indicated as ‘Vouchered soil sample TUE002212 (holotype); eDNA sequence EUK1124409 = OZ253801 (legitype); eDNA sample TUE102212 (nucleotype)’. Typification Details: Typification: TUE 002212. Retrieved: May 19, 2025.

## Additional information

### Artificial Intelligence (AI) use

The authors accept full responsibility for the content of the manuscript, including the disclosure of any use of AI.

Regarding the use of AI in the preparation of this manuscript, the authors declare the following: Gemini.

**Used for**: Figures, tables and charts.

Generative AI was used to assist the design of individual graphic elements used in figures.

### Adherence to national and international regulations

All the fungal strains used in this study have been legally obtained, respecting the Convention on Biological Diversity (Rio Convention).

### Author ORCIDs

V. Fachada https://orcid.org/0000-0002-6575-1943

I.S. Druzhinina https://orcid.org/0000-0003-2821-5268

### Data availability

All of the data that support the findings of this study are available in the main text.

## References

[B1] Abarenkov K, Nilsson RH, Larsson K-H et al. (2024) The UNITE database for molecular identification and taxonomic communication of fungi and other eukaryotes: sequences, taxa and classifications reconsidered. Nucleic Acids Research 52: D791–D797. 10.1093/nar/gkad1039 PMC10767974 37953409

[B2] Abarenkov K, Zirk A, Piirmann T et al. (2025) Full UNITE+INSD dataset for *Fungi*. 10.15156/BIO/3301227

[B3] Aime MC, Miller AN, Aoki T et al. (2021) How to publish a new fungal species, or name, version 3.0. IMA Fungus 12: 11. 10.1186/s43008-021-00063-1 PMC8091500 33934723

[B4] Bernard J (2025) Combining new technology with classic taxonomy to overcome hurdles to discovering dark taxa. Systematics and Biodiversity 23: 2454014. 10.1080/14772000.2025.2454014

[B5] Boettner C, Dayal P, Trebitsch M et al. (2024) Populating the Milky Way: Characterising planet demographics by combining galaxy formation simulations and planet population synthesis models. Astronomy & Astrophysics 686: A167. 10.1051/0004-6361/202449557

[B6] Cassan A, Kubas D, Beaulieu J-P et al. (2012) One or more bound planets per Milky Way star from microlensing observations. Nature 481: 167–169. 10.1038/nature10684 22237108

[B7] Christiansen JL, McElroy DL, Harbut M et al. (2025) The NASA exoplanet archive and exoplanet follow-up observing program: Data, tools, and usage. The Planetary Science Journal 6: 186. 10.3847/PSJ/ade3c2

[B8] Durkin L, Jansson T, Sanchez M et al. (2020) When mycologists describe new species, not all relevant information is provided (clearly enough). MycoKeys 72: 109–128. 10.3897/mycokeys.72.56691 PMC7498475 32982558

[B9] Exoplanet Team [Exoplanet.eu] (2026) Encyclopaedia of exoplanetary systems. [Available from:] https://exoplanet.eu/home/ [January 29, 2026]

[B10] Franz NM (2011) Biological taxonomy and ontology development: scope and limitations. Biodiversity Informatics 7: 45–66. 10.17161/bi.v7i1.3927

[B11] Gannibal PhB (2022) Polyphasic Approach to Fungal Taxonomy. Biology Bulletin Reviews 12: 18–28. 10.1134/S2079086422010029

[B12] Gardes M, Bruns TD (1993) ITS primers with enhanced specificity for basidiomycetes – application to the identification of mycorrhizae and rusts. Molecular Ecology 2: 113–118. 10.1111/j.1365-294X.1993.tb00005.x 8180733

[B13] Gómez-Zapata PA, Díaz-Valderrama JR, Fatemi S et al. (2024) Characterization of the fungal genus *Sphaerellopsis* associated with rust fungi: species diversity, host-specificity, biogeography, and in-vitro mycoparasitic events of *S. macroconidialis* on the southern corn rust, *Puccinia polysora*. IMA Fungus 15: 18. 10.1186/s43008-024-00145-w PMC11223437 38961514

[B14] Grossart H-P, Wurzbacher C, James TY et al. (2016) Discovery of dark matter fungi in aquatic ecosystems demands a reappraisal of the phylogeny and ecology of zoosporic fungi. Fungal Ecology 19: 28–38. 10.1016/j.funeco.2015.06.004

[B15] Hawksworth DL (2003) The lichenicolous fungi of Great Britain and Ireland: an overview and annotated checklist. The Lichenologist 35: 191–232. 10.1016/S0024-2829(03)00027-6

[B16] Hawksworth DL, Lücking R (2017) Fungal Diversity Revisited: 2.2 to 3.8 Million Species. Heitman J, James TY (Eds). Microbiology Spectrum 5: 5.4.10. 10.1128/microbiolspec.FUNK-0052-2016 PMC11687528 28752818

[B17] Hibbett DS, Taylor JW (2013) Fungal systematics: is a new age of enlightenment at hand? Nature Reviews Microbiology 11: 129–133. 10.1038/nrmicro2963 23288349

[B18] Hibbett DS, Ohman A, Glotzer D et al. (2011) Progress in molecular and morphological taxon discovery in *Fungi* and options for formal classification of environmental sequences. Fungal Biology Reviews 25: 38–47. 10.1016/j.fbr.2011.01.001

[B19] Hyde KD, Jeewon R, Chen Y-J et al. (2020) The numbers of fungi: is the descriptive curve flattening? Fungal Diversity 103: 219–271. 10.1007/s13225-020-00458-2

[B20] Jones MDM, Richards TA, Hawksworth DL et al. (2011) Validation and justification of the phylum name *Cryptomycota* phyl. nov. IMA Fungus 2: 173–175. 10.5598/imafungus.2011.02.02.08 PMC3359815 22679602

[B21] Kim J (2024) Fungal identification based on the polyphasic approach: a clinical practice guideline. Annals of Clinical Microbiology 27: 221–230. 10.5145/ACM.2024.27.4.2

[B22] Kirk PM, Griffith GW (2021) Nomenclatural novelties. Index Fungorum 467: 1.

[B23] Lehtonen S, Thiele KR (2023) Report of the Special‐purpose Committee on DNA Sequences as Types, established at the XIX International Botanical Congress in Shenzhen, China. TAXON 72: 1137–1142. 10.1002/tax.13055

[B24] Li QR, Pi YH, Charria-Girón E et al. (2025) Polyphasic taxonomy and chemical diversity of *Annulohypoxylon* (*Ascomycota*, *Hypoxylaceae*): New species from China’s tropical forest. Persoonia – Molecular Phylogeny and Evolution of Fungi 55: 159–202. 10.3114/persoonia.2025.55.05 PMC12798846 41536597

[B25] Lofgren LA, Stajich JE (2021) Fungal biodiversity and conservation mycology in light of new technology, big data, and changing attitudes. Current Biology 31: R1312–R1325. 10.1016/j.cub.2021.06.083 PMC8516061 34637742

[B26] Lücking R, Moncada B (2017) Dismantling *Marchandiomphalina* into *Agonimia (Verrucariaceae)* and *Lawreymyces* gen. nov. (*Corticiaceae*): setting a precedent to the formal recognition of thousands of voucherless fungi based on type sequences. Fungal Diversity 84: 119–138. 10.1007/s13225-017-0382-4

[B27] Lücking R, Hawksworth DL (2018) Formal description of sequence-based voucherless *Fungi*: promises and pitfalls, and how to resolve them. IMA Fungus 9: 143–165. 10.5598/imafungus.2018.09.01.09 PMC6048566 30018876

[B28] Lücking R, Kirk PM, Hawksworth DL (2018) Sequence-based nomenclature: a reply to Thines et al. and Zamora et al. and provisions for an amended proposal “from the floor” to allow DNA sequences as types of names. IMA Fungus 9: 185–198. 10.5598/imafungus.2018.09.01.12 PMC6048568 30018879

[B29] Lücking R, Aime MC, Robbertse B et al. (2020) Unambiguous identification of fungi: where do we stand and how accurate and precise is fungal DNA barcoding? IMA Fungus 11: 14. 10.1186/s43008-020-00033-z PMC7353689 32714773

[B30] Lücking R, Aime MC, Robbertse B et al. (2021) Fungal taxonomy and sequence-based nomenclature. Nature Microbiology 6: 540–548. 10.1038/s41564-021-00888-x PMC10116568 33903746

[B31] May TW (2020) Procedures and timetable for proposals to amend Chapter F of the International Code of Nomenclature for algae, fungi, and plants. IMA Fungus 11: 21. 10.1186/s43008-020-00044-w PMC7513284 33014692

[B32] May TW, Miller AN (2018) International Mycological Congress: Guiding Vote on nomenclature proposals to amend Chapter F of the International Code of Nomenclature for algae, fungi, and plants. IMA Fungus 9: xv–xxi. 10.1007/BF03449447 PMC7325661 32647625

[B33] May TW, Hawksworth DL (2024) Proposals for consideration at IMC12 to modify provisions related solely to fungi in Chapter F of the International Code of Nomenclature for algae, fungi, and plants. IMA Fungus 15: 25. 10.1186/s43008-024-00152-x PMC11323459 39143648

[B34] May TW, Bensch K (2024) Synopsis of proposals on fungal nomenclature: a review of the proposals concerning Chapter F of the International Code of Nomenclature for algae, fungi, and plants submitted to the XII International Mycological Congress, 2024. IMA Fungus 15: 26. 10.1186/s43008-024-00151-y PMC11330136 39152513

[B35] May TW, Bensch K (2026) Procedures and timetable for proposals to amend Chapter F of the International Code of Nomenclature for algae, fungi, and plants. IMA Fungus 17: e195573. 10.3897/imafungus.17.195573 PMC13266449 42306629

[B36] May TW, Redhead SA, Bensch K et al. (2019) Chapter F of the International Code of Nomenclature for algae, fungi, and plants as approved by the 11^th^ International Mycological Congress, San Juan, Puerto Rico, July 2018. IMA Fungus 10: 21. 10.1186/s43008-019-0019-1 PMC7325661 32647625

[B37] May TW, Bensch K, Groenewald JZ et al. (2024) XII International Mycological Congress: report of Congress action on nomenclature proposals relating to fungi. IMA Fungus 15: 36. 10.1186/s43008-024-00169-2 PMC11580392 39574204

[B38] McNeill J, Barrie FR, Buck WR et al. [Eds] (2012) International code of nomenclature for algae, fungi and plants (Melbourne code): adopted by the Eighteenth International Botanical Congress, Melbourne, Australia, July 2011. Koeltz Scientific Books, Königstein, 208 pp.

[B39] NASA (2026) Cumulative number of exoplanets discovered, by method. [Available from:] https://archive.ourworldindata.org/20260109-090050/grapher/cumulative-exoplanets-by-method.html [January 29, 2026]

[B40] NASA Exoplanet Science Institute (2020) Planetary Systems Table. 10.26133/NEA12

[B41] Nilsson RH, Ryberg M, Wurzbacher C et al. (2023) How, not if, is the question mycologists should be asking about DNA-based typification. MycoKeys 96: 143–157. 10.3897/mycokeys.96.102669 PMC10194844 37214179

[B42] Robert V, Vu D, Amor ABH et al. (2013) MycoBank gearing up for new horizons. IMA Fungus 4: 371–379. 10.5598/imafungus.2013.04.02.16 PMC3905949 24563843

[B43] Ryberg M, Nilsson RH (2018) New light on names and naming of dark taxa. MycoKeys 30: 31–39. 10.3897/mycokeys.30.24376 PMC5904500 29681731

[B44] Schadt CW, Martin AP, Lipson DA et al. (2003) Seasonal dynamics of previously unknown fungal lineages in tundra soils. Science 301: 1359–1361. 10.1126/science.1086940 12958355

[B45] Selbmann L, Benkő Z, Coleine C et al. (2020) Shed Light in the DaRk LineagES of the Fungal Tree of Life—STRES. Life 10: 362. 10.3390/life10120362 PMC7767062 33352712

[B46] Simões ARG, Leliaert F, Bramley GLC et al. (2025) Equipping the next generation of plant taxonomists: Insights and recommendations. Trends in Plant Science: 31: P677–691. 10.1016/j.tplants.2025.08.019 41198420

[B47] Tedersoo L, Bahram M, Põlme S et al. (2014) Global diversity and geography of soil fungi. Science 346: 1256688. 10.1126/science.1256688 25430773

[B48] Tedersoo L, Hosseyni Moghadam MS, Panksep K et al. (2025) Thirty novel fungal lineages: formal description based on environmental samples and DNA. MycoKeys 124: 1–121. 10.3897/mycokeys.124.161674 PMC12559954 41163701

[B49] Thiele KR, Groom Q, Renner SS et al. (2023) (329–338) Proposals to permit DNA sequences to serve as types of names in prescribed circumstances. TAXON 72: 1143–1145. 10.1002/tax.13031

[B50] Thines M, Crous PW, Aime MC et al. (2018) Ten reasons why a sequence-based nomenclature is not useful for fungi anytime soon. IMA Fungus 9: 177–183. 10.5598/imafungus.2018.09.01.11 PMC6048572 30018878

[B51] Thomson SA, Pyle RL, Ahyong ST et al. (2018) Taxonomy based on science is necessary for global conservation. PLOS Biology 16: e2005075. 10.1371/journal.pbio.2005075 PMC5851535 29538381

[B52] Tripp EA, Lendemer JC (2012) (4–5) Request for binding decisions on the descriptive statements associated with *Mortierella sigyensis* (fungi: *Mortierellaceae*) and *Piromyces cryptodigmaticus* (fungi: *Neocallimastigaceae*). TAXON 61: 886–888. 10.1002/tax.614022

[B53] Turland N, Wiersema J, Barrie F et al. [Eds] (2018) 159 International Code of Nomenclature for algae, fungi, and plants. Koeltz Botanical Books. 10.12705/Code.2018

[B54] Turland N, Wiersema JH, Barrie FR et al. [Eds] (2025) International Code of Nomenclature for Algae, Fungi, and Plants (Madrid Code). 1^st^ ed. University of Chicago Press, Chicago, 1 pp.

[B55] Turland NJ (2025) From the Shenzhen Code to the Madrid Code: New rules and recommendations for naming algae, fungi, and plants. American Journal of Botany 112: e70026. 10.1002/ajb2.70026 PMC12012794 40186357

[B56] Turland NJ, Wiersema JH (2024) Synopsis of Proposals on Nomenclature – Madrid 2024: A review of the proposals to amend the International Code of Nomenclature for algae, fungi, and plants submitted to the XX International Botanical Congress. TAXON 73: 325–404. 10.1002/tax.13114

[B57] Turland NJ, Kempa M, Knapp S et al. (2024a) Results of the preliminary guiding vote (“mail vote”) on proposals to amend the International Code of Nomenclature for algae, fungi, and plants submitted to the XX International Botanical Congress, Madrid 2024. TAXON 73: 1096–1109. 10.1002/tax.13233

[B58] Turland NJ, Álvarez I, Knapp S et al. (2024b) XX International Botanical Congress, Madrid 2024: Report of Congress action on nomenclature proposals. TAXON 73: 1308–1323. 10.1002/tax.13258

[B59] Turland NJ, Wiersema JH, Gravendyck J (2026) Instructions for authors of proposals to amend the International Code of Nomenclature for algae, fungi, and plants. TAXON 75: e70126. 10.1002/tax.70126

[B60] Větrovský T, Morais D, Kohout P et al. (2020) GlobalFungi, a global database of fungal occurrences from high-throughput-sequencing metabarcoding studies. Scientific Data 7: 228. 10.1038/s41597-020-0567-7 PMC7359306 32661237

[B61] Wu B, Hussain M, Zhang W et al. (2019) Current insights into fungal species diversity and perspective on naming the environmental DNA sequences of fungi. Mycology 10: 127–140. 10.1080/21501203.2019.1614106 PMC6691916 31448147

[B62] Yurkov A, Visagie CM, Crous PW et al. (2024) Cultures as types and the utility of viable specimens for fungal nomenclature. IMA Fungus 15: 20. 10.1186/s43008-024-00155-8 PMC11267820 39049113

[B63] Zamora JC, Svensson M, Kirschner R et al. (2018) Considerations and consequences of allowing DNA sequence data as types of fungal taxa. IMA Fungus 9: 167–175. 10.5598/imafungus.2018.09.01.10 PMC6048565 30018877

[B64] Zhang Z-F, Jiang Y, Mao J (2025) Global distribution patterns of dark matter fungi in cold seep: A metagenomic meta-analysis. Journal of Fungi 11: 878. 10.3390/jof11120878 PMC12734366 41440703

[B65] Zhou L-W (2024) The strategy for naming fungal ‘dark taxa’ may involve a transition period and genomics. Fungal Biology Reviews 48: 100358. 10.1016/j.fbr.2024.100358

